# 1795. Exploring the role of reported sexual contact in mpox cases in California, May - October, 2022

**DOI:** 10.1093/ofid/ofad500.1624

**Published:** 2023-11-27

**Authors:** Elissa Kim, Kayla Saadeh

**Affiliations:** CSTE/CDPH, Hayward, California; California Department of Public Health, Richmond, California

## Abstract

**Background:**

An estimated 94% of mpox cases in the United States have been acquired via male-to-male sexual contact, but non-sexual transmission has been documented. We compared demographics, clinical characteristics, and exposure information among mpox cases in California who did and did not report sexual exposure (SE) in the 21 days prior to infection.

**Methods:**

All mpox cases reported in California from May - Oct 2022 were eligible for inclusion. SE was defined as reported sexual engagement and/or close intimate contact with another person within 21 days prior to symptom onset or having a reported first rash/lesion in the genital, perianal, or groin areas. Cases who neither reported SE nor first rash/lesion in those areas were defined as cases without SE. Cases with missing SE history were excluded. The California Healthy Places Index (HPI), a geospatial equity index, was used to evaluate social determinants of health in respondents’ communities of residence.

**Results:**

Of 5278 cases reported in the study period, 4201 (79.6%) reported SE history data. Of those, 3661 (87.1%) reported SE (133 (3.6%) were identified by lesion location). A higher proportion of females (n=22, 22.9%, p< 0.01) reported no SE compared to other gender identities. A higher proportion of Black/African American (n=93, 18.6%, p< 0.0001) and Hispanic/Latino (n=280, 16.3%, p=0.0002) reported no SE compared to White (n=110, 8.6%, p< 0.0001). A higher proportion of straight/heterosexual persons (n=100, 29.9%, p< 0.0001) reported no SE compared to gay/lesbian persons (n=303, 10.0%, p< 0.0001). Persons in HPI Q1 (least opportunities to lead healthy lives) had the highest percentage reporting no SE (n=40, 11.4%, p< 0.001), while Q3 and Q4 (most opportunities to lead healthy lives) had the lowest SE reporting (n=27, 5.5% and n=42, 5.6%, respectively).

**Figure:**
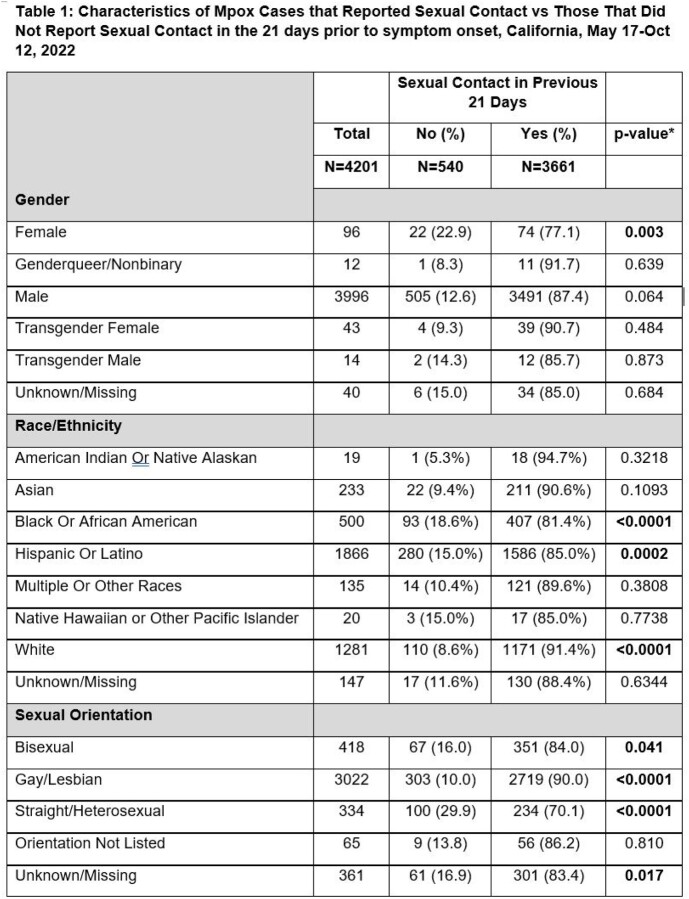

Table 1

**Figure:**
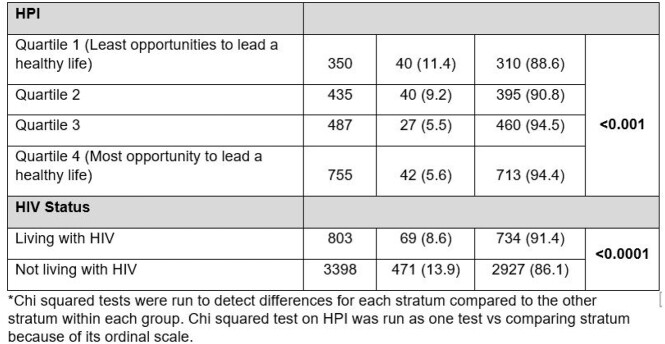

Characteristics of Mpox Cases that Reported Sexual Contact vs Those That Did Not Report Sexual Contact in the 21 days prior to symptom onset, California, May 17-Oct 12, 2022

**Conclusion:**

Females reporting Black/African American or Hispanic/Latino race/ethnicity identifying as straight/heterosexual, and persons living in HPI Q1 were less likely to report SE than their counterparts. Factors that may influence one’s willingness to disclose SE were not measured in this analysis. Nonetheless, variation in report of SE among confirmed mpox cases indicates that providers should consider mpox when clinically relevant, even in cases without reported SE.

**Disclosures:**

**All Authors**: No reported disclosures

